# Gastric Adenocarcinoma Presenting with Gastric Outlet Obstruction in a Child

**DOI:** 10.1155/2014/527471

**Published:** 2014-01-14

**Authors:** Abdulrahman Al-Hussaini, Salem AlGhamdi, Rasha Alsaaran, Fawaz Al-Kasim, Zakaria Habib, Nouri Ourfali

**Affiliations:** ^1^Division of Pediatric Gastroenterology, Hepatology & Nutrition, Children's Hospital, University of King Saud for Health Sciences, King Fahad Medical City, P.O. Box 59046, Riyadh 11525, Saudi Arabia; ^2^Department of Pediatrics, College of Medicine, King Saud University and King Khalid University Hospital, Riyadh, Saudi Arabia; ^3^Oncology/Hematology Division, King Saud Medical City, P.O. Box 58594, Riyadh 11515, Saudi Arabia; ^4^King Faisal Specialist Hospital and Research Center, Department of Surgery, P.O. Box 3354, Riyadh 11211, Saudi Arabia

## Abstract

Gastric carcinoma is extremely rare in children representing only 0.05% of all gastrointestinal malignancies. Here, we report the first pediatric case of gastric cancer presenting with gastric outlet obstruction. Upper endoscopy revealed a markedly thickened antral mucosa occluding the pylorus and a clean base ulcer 1.5 cm × 2 cm at the lesser curvature of the stomach. The narrowed antrum and pylorus underwent balloon dilation, and biopsy from the antrum showed evidence of *Helicobacter pylori* gastritis. The biopsy taken from the edge of the gastric ulcer demonstrated signet-ring-cell type infiltrate consistent with gastric adenocarcinoma. At laparotomy, there were metastases to the liver, head of pancreas, and mesenteric lymph nodes. Therefore, the gastric carcinoma was deemed unresectable. The patient died few months after initiation of chemotherapy due to advanced malignancy. In conclusion, this case report underscores the possibility of gastric adenocarcinoma occurring in children and presenting with gastric outlet obstruction.

## 1. Introduction

Malignancies of the gastrointestinal tract are very rare in children, accounting for 1.2% of pediatric malignancies [1]. Malignant gastric tumors that affect children are mostly lymphomas and soft-tissue sarcomas. Gastric carcinomas are extremely rare in children, accounting for 0.05% of gastrointestinal malignancies [2]. Because of the rarity of gastric carcinoma in children and nonspecificity of presenting symptoms, the diagnosis is usually made late and consequently the prognosis is poor due to established metastases by the time of diagnosis. Here, we report a child with gastric adenocarcinoma and a unique presentation of gastric outlet obstruction. We aim to increase awareness for this type of tumor and from that to improve the prognosis in pediatric patients.

## 2. Case Report

**Figure 1:**
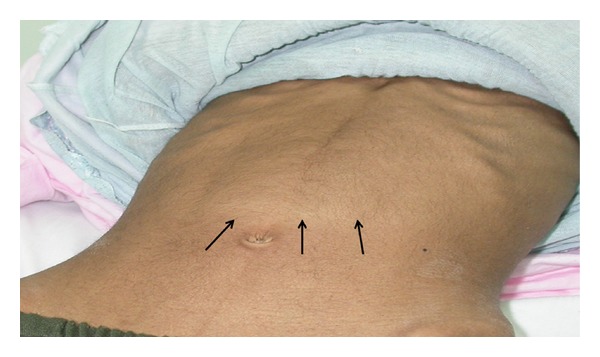
Fullness at epigastrium due to gastric distension.

A 10-year-old Saudi girl was referred to our hospital with a complaint of progressive intermittent vomiting for 6 months. The vomiting was projectile of semidigested food content and almost always after meals. It was associated with nonspecific nonradiating epigastric pain, undocumented weight loss, progressive fatigability, and bone aches. There was no history of fever, loose bowel motions, or hematemesis. Her family history was negative for tuberculosis, peptic ulcer diseases, or gastric carcinoma. At physical examination, she was emaciated, sick, pale, and severely dehydrated. There were no palpable pathologic lymph nodes, jaundice, or skin rash. She was afebrile with pulse rate 110 beats/minute, respiratory rate 20 breaths/minute, and blood pressure 90/60 mmHg. Her weight was 18.9 kg (<5th percentile for age and sex) and height 137.5 cm (on the 50th percentile). Local examination revealed a soft and lax abdomen with fullness at epigastrium (Figure 1) with no palpable masses, organomegaly, or ascites. Another systemic examination was unremarkable.

**Figure 2:**
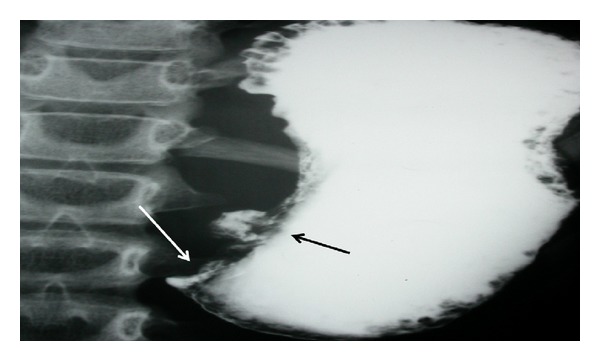
Barium meal shows obstruction at pylorus (white arrow) and a crater of an ulcer (black arrow).

Initial laboratory investigations showed hemoglobin 14 gm/dL, white blood cell count 9.2 × 10^9^/L, platelet count 459 × 10^3^/mm^3^, erythrocyte sedimentation rate 30 mm/hr (normal, 0–10 mm/hr), and normal peripheral blood smear. Her blood gases revealed pH 7.59, PCO_2_ 50 kPa, and HCO_3_ 55 mmol/L. Other laboratory workup showed hyponatremia (122 mmol/L), hypokalemia (1.87 mmol/L), hypochloremia (62 mmol/L), hyperuricemia (418 mmol/L), and elevated urea (13 mmol/L) and creatinine (69 mmol/L). Her liver function test, serum amylase/lipase and lactate dehydrogenase levels were all normal. At this stage, the clinical diagnosis was gastric outlet obstruction. After correction of dehydration and electrolyte imbalance and establishment of total parenteral nutrition, she underwent barium meal which confirmed the clinical impression of gastric outlet obstruction and in addition showed a crater of an ulcer at lesser curvature (Figure 2).

**Figure 3:**
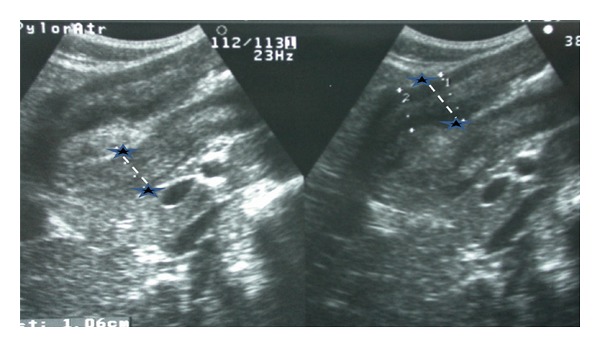
Ultrasound abdomen shows thickening of antral (asterisks) and pyloric wall.

**Figure 4:**
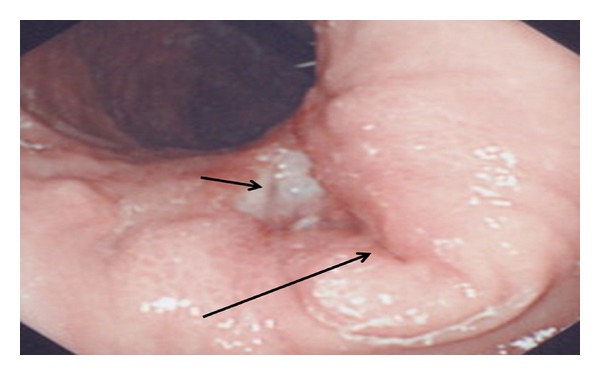
Endoscopic view of obstruction at pylorus (long arrow) and a clean bed of an ulcer at lesser curvature (short arrow).

**Figure 5:**
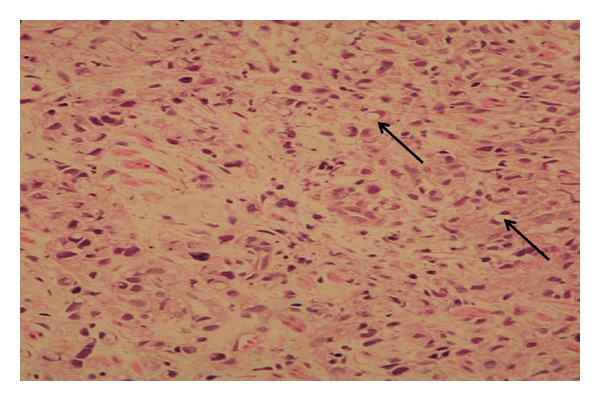
Histopathology of a biopsy form edge of the gastric ulcer shows signet-ring-cell infiltrate (arrows) consistent with gastric adenocarcinoma. (Hematoxylin and eosin staining; magnification ×40).

Ultrasound of abdomen revealed marked thickening of antral wall (12 mm in diameter) and pyloric channel (Figure 3) and moderate amount of fluid within the pelvis. Upper gastrointestinal endoscopy showed a markedly erythematous, fragile and erosive mucosa at lower esophagus, a markedly thickened antral mucosa occluding the pylorus, and a clean base ulcer 2 centimeters × 1.5 centimeters at the lesser curvature of the stomach (Figure 4). The narrowed antrum and pylorus underwent balloon dilatation; afterwards, it was possible to pass pediatric size scope (8.6 mm) through the pylorus down to the duodenum which looked normal and nasojejunal tube was passed through for continuous enteral feeding. The esophageal biopsy showed reflux esophagitis, and the antral biopsies revealed active chronic *Helicobacter pylori *gastritis grade IV with no metaplastic changes. The biopsies obtained from the edge of the gastric ulcer demonstrated poorly differentiated signet-ring-cell adenocarcinoma (Figure 5). For staging purposes, abdominal CT scan was performed. It revealed marked thickening of antral wall (18 mm in diameter) and pyloric channel and hypodense soft tissue densities at the celiac axis suggestive of celiac lymphadenopathy, and no definite signs of infiltration either of the liver or pancreas could be visualized. CT scan of the chest showed normal lungs, heart, and pleural spaces and no evidence of metastatic lesions or lymphadenopathy. Bone scan study was negative for evidence of metastases.

The child underwent exploratory laparotomy to evaluate for resectability of the gastric tumor. There were small metastases to the liver, head of pancreas, and mesenteric lymph nodes and aspirate from ascitic fluid revealed malignant cells. Therefore, the gastric carcinoma was deemed unresectable and a gastrojejunostomy tube was placed for enteral nutrition and gastric decompression. The patient received chemotherapy consisting of cisplatin, 5-fluorouracil, and methotrexate. Followup CT scan of abdomen showed no response, so the patient was put on palliative care including analgesics. Unfortunately, she died few months after diagnosis due to advanced carcinoma.

## 3. Discussion

Gastric carcinoma primarily affects patients in the 50-to-70-year age group and is uncommon before the fifth decade of life. Worldwide, there is a large geographic difference in gastric cancer incidence. According to statistics from the International Agency for Research on Cancer, about one million new cases of stomach cancer were estimated to have occurred in 2008 (7.8% of the total), making it currently the fourth most common malignancy in the world, behind cancers of the lung, breast, and colorectum [3]. More than 70% of cases (713 000 cases) occur in developing countries and half the world total occurs in Eastern Asia (mainly in China and Japan). Age-standardized incidence rates (ASR) are about twice as high in men as in women, ranging from 3.9 in Northern Africa to 42.4 in Eastern Asia for men and from 2.2 in Southern Africa to 18.3 in Eastern Asia for women. On the contrary, primary gastric carcinoma is extremely rare in pediatric age group. Our review of medical literature since 1966 revealed that around 30 cases of gastric carcinoma have been reported in children or adolescents.

The 2010 Tumor Registry annual report of the Oncology Center at King Faisal Specialist Hospital and Research Center in Riyadh, Saudi Arabia, showed that a total of 561 cases of gastric cancer were diagnosed over 10-year-period from 2001 to 2010 [4]. Only 2 pediatric cases were diagnosed with gastric cancer during the same period (0.0035%).

The rarity of gastric cancer in pediatric age group, unawareness of pediatricians about the possibility of gastric carcinoma in children, and nonspecificity of initial gastrointestinal symptoms led to significant delay in diagnosis and late initiation of appropriate medical therapy and surgical management. Consequently, gastric cancer in children is a more advanced case on presentation and associated with a worse prognosis than adults. The reported 5-year disease-free survival rate in adults is approximately 15% [5]. Among the pediatric cases reported in the literature, median survival after diagnosis is approximately 5 months. The only 2 long-term survivors free of disease, 30 and 102 months after surgical resection [6, 7], had localized disease that had complete surgical removal of primary tumor. The onset of hematemesis in one case [6] was an alarming symptom that led to early consideration of upper endoscopy and biopsies and early diagnosis of ulcerative gastric cancer.

Symptoms of gastric carcinoma vary according to the location and extent of the tumor, with tumor arising at cardia of the stomach presenting with dysphagia [8] and tumors distal to the cardia are manifesting nonspecific gastrointestinal symptoms like abdominal pain, loss of appetite, weight loss, vomiting, heart burn, anorexia, fatigue, and malaise [6]. Our patient is the first pediatric case to present with gastric outlet obstruction. Other differential diagnoses of gastric outlet obstruction that need to be considered include infectious cause (brucellosis, fungal infection, or tuberculosis), cicatricial gastric ulcer/duodenal ulcer, gastric bezoars, antral web, neoplastic lesions (lymphoma), and inflammatory lesions including Crohn's disease, eosinophilic gastritis, and chronic granulomatous disease [9].

The etiology of gastric cancer has been demonstrated to be multifactorial, infection with *Helicobacter pylori* being the most important risk factor [10]. Virulence of *Helicobacter pylori* depends on several factors including the cytotoxic associated gene A and the vacuolating cytotoxin gene A [11]. Whether the existence of *Helicobacter pylori *infection and gastric cancer in our patient is of causal or concurrent relationship remains unclear. Occurrence of gastric cancer in young people motivated molecular studies that identified inherited mutations in the E-cadherin/CDH1 gene described in several ethnic groups [12–14].

In conclusion, gastric carcinoma in children is extremely rare. This case report underscores the possibility of gastric carcinoma occurring in children and should be considered in the differential diagnosis of gastric outlet obstruction. The diagnosis should be made at earlier stages, to provide better chances for these patients to undergo curative treatment.
